# Successful Treatment of Cutaneous *Curvularia geniculata, Nocardia niigatensis*, and Viral Papillomatosis in a Dog During the Therapeutic Management of Immune-Mediated Hemolytic Anemia

**DOI:** 10.3389/fvets.2019.00249

**Published:** 2019-08-08

**Authors:** Emily Strzok, Chris Siepker, Abigail Armwood, Elizabeth Howerth, Joanne Smith, Frane Banovic

**Affiliations:** ^1^Department of Small Animal Medicine and Surgery, College of Veterinary Medicine, University of Georgia, Athens, GA, United States; ^2^Department of Pathology, College of Veterinary Medicine, University of Georgia, Athens, GA, United States

**Keywords:** opportunistic skin infections, canine, skin, dermatology, immunosuppression

## Abstract

Opportunistic infections represent a major cause of mortality in immunocompromised patients. Discontinuation or reduction of immunosuppressive medications is recommended with the development of opportunistic infections, which may cause a relapse or worsening of the immune-mediated disease. A 7.5-year-old, spayed female great Dane was diagnosed with immune-mediated hemolytic anemia with initial immunosuppressive therapy consisting of oral prednisone, ciclosporin and mycophenolate mofetil. The patient developed diffuse right forelimb pyogranulomatous fungal dermatitis with deep draining tracts 6 weeks into immunosuppressive treatment with *Curvularia geniculata* growth. Oral once daily terbinafine and itraconazole were initiated; ciclosporin was immediately discontinued and the mycophenolate mofetil/prednisone doses were reduced. The right forelimb skin lesions resolved after 4 weeks, but the patient presented with a diffuse severe neutrophilic dermatitis on the left forelimb; 16S rRNA sequencing identified *Nocardia niigatensis*. Cutaneous nocardiosis was treated with oral enrofloxacin and doxycycline; systemic immunosuppressive therapies were continued for immune-mediated hemolytic anemia control. One month later, the left forelimb lesions completely resolved but the patient developed several multifocal, exophytic warts; the clinical features and histopathology were consistent with viral papillomas. Within the following 4 weeks, the patient developed severe diffuse papillomatosis of the left forelimb, which was successfully treated with 2 weeks of every other day topical imiquimod administration. In this case, successful treatment of cutaneous opportunistic bacterial, fungal and viral infection was possible with proper treatment even though the immunosuppressive drug treatments could not be discontinued.

## Background

Opportunistic infections represent a major cause of mortality in immunocompromised patients and are becoming more prevalent with the increased use of single and multi-agent immunosuppressive medications for treatment of immune-mediated and autoimmune diseases. Reports of opportunistic cutaneous infections in dogs associated with immunosuppression include *Nocardia* species (1), saprophytic fungi (phaeohyphomycosis and hyalohyphomycosis) (2, 3) and viral-induced papillomas (4, 5). Discontinuation of immunosuppressive medications is recommended with the development of opportunistic infections, which may cause a relapse or worsening of the immune-mediated disease (3, 6).

Herein, we describe successful treatment of cutaneous *Curvularia geniculata, Nocardia niigatensis* and viral papillomatosis with continued immunosuppression for treatment of immune-mediated hemolytic anemia (IMHA).

## Case Presentation

A 7.5-year-old, 62 kg, female spayed Great Dane was referred for lethargy and anorexia. The initial complete blood count (CBC) and chemistry panel revealed a regenerative anemia with hyperbilirubinemia. Repeat CBC and chemistry profile revealed progressive regenerative anemia with spherocytosis and worsening hyperbilirubinemia confirming the diagnosis of IMHA. A comprehensive diagnostic IMHA work-up was performed to identify a possible underlying cause. A comprehensive tick panel, blood culture and urine culture were negative. A thoracic and abdominal CT scan did not identify an underlying cause. Three days later, the patient was started on oral twice daily mycophenolate mofetil (12 mg/kg AM and 8 mg/kg PM, MMF; Ascend Lab, LLC, Parsippany, NJ, USA). Due to worsening condition, the patient was hospitalized, received intravenous immunosuppression with MMF and dexamethasone, and received several multiple packed red blood cell transfusions. Due to lack of response to immunosuppressive therapies, a splenectomy was performed 2 weeks after initial presentation; histopathology report revealed fibrosis, congestion, hemosiderosis, and extramedullary hematopoiesis consistent with IMHA. Prior to discharge, about 3 weeks from initial presentation, the patient's IMHA treatment included oral twice daily MMF (12 mg/kg AM and 8 mg/kg PM), prednisone (0.5 mg/kg; West-ward Pharmaceuticals, Eatontown, NJ, USA), and ciclosporin (5 mg/kg; Elanco, Greenfield, NJ, USA).

**Figure 1:**
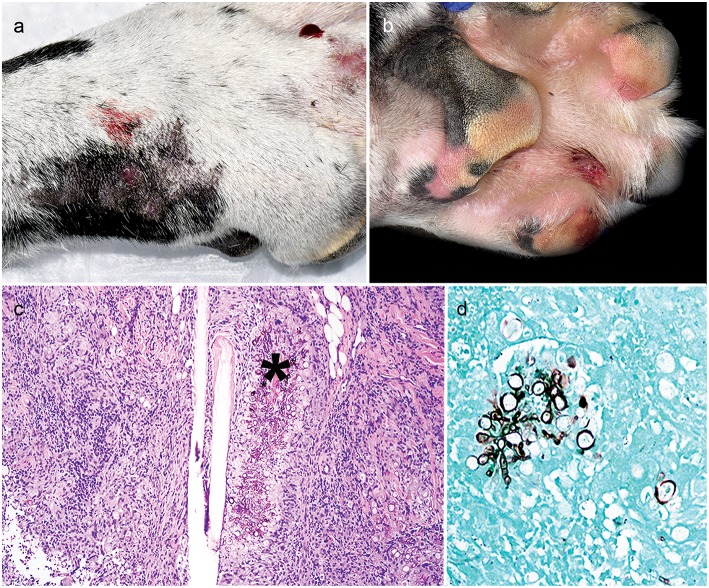
Clinical and histopathology appearance of cutaneous *Curvularia geniculata*. Right forelimb paw swelling with multifocal draining tracts **(a,b)**; Note pyogranulomatous dermatitis with intralesional pigmented fungal elements (asterisk) centered around hair follicles and adnexa (**c**: 10X, H&E stain); Note the GMS-positive septate fungal elements, which form large, terminal dilations (**d**: 20X, GMS stain).

The patient presented just over 6 weeks after initial referral with right forelimb paw swelling and multifocal draining tracts (Figures 1a,b). With the patient receiving three immunosuppressive medications, differentials included an opportunistic fungal infection or bacterial infection. Skin cytology showed marked pyogranulomatous inflammation with non-staining, septate fungal hyphae. Punch biopsies of ulcerated skin lesions were submitted for fungal culture and routinely processed in paraffin wax and stained with hematoxylin and eosin (H&E) and Gomorimethenamine silver (GMS) fungal stain. Examination of H&E stained biopsies showed pyogranulomatous dermatitis with intralesional GMS-positive pigmented fungal elements, centered around hair follicles and adnexa, consistent with phaeohyphomycosis (Figures 1c,d). Fungal culture grew a pigmented fungus and polymerase chain reaction (PCR) utilizing primers of internal transcribed spacer (ITS) D1/D2 regions from extracted fungal culture DNA produced a 1,085 bp band. The band sequencing and analysis using BLASTn search in GenBank revealed 99% sequence homology with *Curvularia geniculata*. Antifungal therapy was initiated with once daily oral terbinafine (32 mg/kg; Cipla USA, Sunrise, FL, USA) and itraconazole (5 mg/kg; PAR, Chestnut Ridge, NY, USA). The administration of immunosuppressive medications was modified; ciclosporin was immediately discontinued, prednisone was decreased (0.5 mg/kg AM and 0.3 mg/kg PM) and MMF lowered to once daily (12 mg/kg).

**Figure 2:**
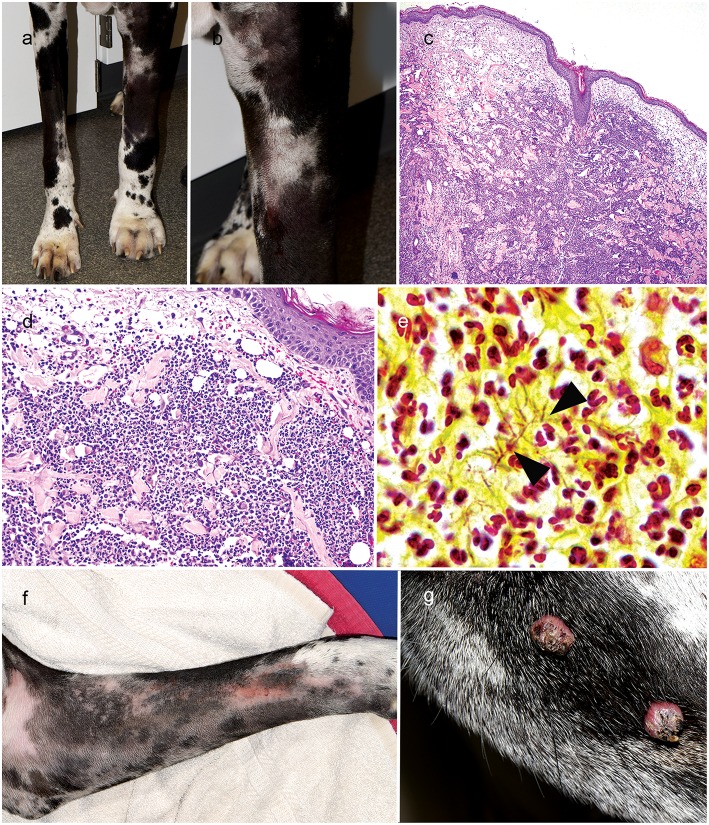
Right forelimb improvement after treatment of *Curvularia geniculata* **(a)**; Clinical appearance of cutaneous *Nocardia niigatensis* with diffuse left forelimb edema, nodules and two draining tracts **(a, b)**; Histopathology of cutaneous nocardiosis is characterized by diffuse neutrophilic dermatitis with superficial dermal edema and small numbers of macrophages(**c**: 4X, H&E stain; **d**: 20X, H&E stain); Rare, slender gram-positive branching bacilli (arrowheads) scattered throughout the deep dermis with neutrophilic cell infiltration(**e**: 100X, Gram-stain); Clinical improvement of left forelimb after treatment of cutaneous Nocardiosis **(f)**; Multifocal exophytic cutaneous viral papillomason lateral muzzle **(g)**.

On progress examination, 4 weeks later (10 weeks from initial presentation), the right forelimb skin lesions completely resolved (Figure 2a), but the patient developed acute diffuse upper left forelimb edema, nodules, and two draining tracts (Figures 2a,b). Fine needle aspirate showed marked pyogranulomatous inflammation with rare multinucleated giant cells; there were no structures suggestive of bacterial or fungal infection. Skin biopsies were obtained and routinely processed in paraffin wax and stained with H&E, Gram, Giemsa, modified Ziehl–Neelsen (ZN), periodic acid–Schiff (PAS), and Warthin-Starry silver stain; fresh tissue from the affected lesional skin was submitted for fungal and bacterial aerobicculture. Histopathologic examination revealed marked diffuse neutrophilic dermatitis with superficial dermal edema and small numbers of macrophages (Figures 2c,d). Argyrophilic, slender Gram- and Giemsa-stain positive branching bacilli were scattered throughout the deep dermis with neutrophilic infiltration (Figure 2e); PAS and modified ZN stains were negative.

Aerobic bacterial culture of the fresh tissue yielded branching gram-positive bacilli and *Streptomyces* spp. was identified as the causative pathogen by conventional phenotypic bacterial identification methods as well as matrix-absorption laser desorption ionization-time-of-flight mass spectrometry (MALDI-TOF MS). The *Streptomyces* spp. isolate was sensitive to amikacin, trimethoprim-sulfamethoxazole (TMS), and moxifloxacin. *Streptomyces* infection in dogs are rare with concern for a bacterial contaminant (7). The isolate was subjected to 16S rRNA sequencing using published primers (8), followed by direct Sanger sequencing of PCR product and GenBankBLASTn searches. Results confirmed the amplified 1,373 bp band sequences to be 100% homologous to the *Nocardia niigatensis* complete 16S rRNA sequence (AB092563). Treatment was initiated with once daily oral enrofloxacin (5 mg/kg; Bayer, Shawnee Mission, KA, USA) and twice daily doxycycline (5 mg/kg; West-Ward Pharmaceuticals, Eatontown, NJ, USA); immunosuppressants MMF (12 mg/kg oncedaily) and prednisone (0.5 mg/kg AM and 0.3 mg/kg PM) were continued.

**Figure 3:**
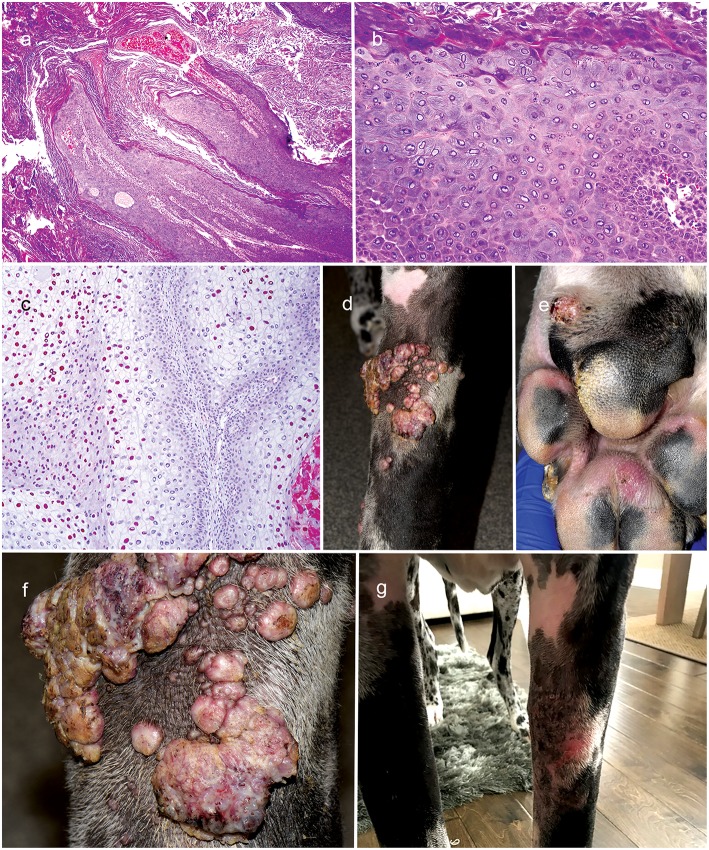
Clinical appearance and histopathology of cutaneous viral exophyticpapillomas. Note the papillary hyperplasia and severe hyperkeratotis (**a**: 4X, H&Estain). Keratinocytes are enlarged with gray-blue finely fibrillar cytoplasm and sometimes have a large amphophilic, intranuclear inclusion body that peripheralizes the chromatin (**b**: 20X, H&E stain); Immunohistochemistry staining with anti-human papillomavirus antibodies reveals the presence of papillomaviral L1 positive for viral papillomas (**c**: 10X; broad spectrum BPV-1/IH8 and CAMVIR-1 antibody). Diffuse to coalescing viral-papillomas on the left forelimb limb and margin of paw pad **(d–f)**; Near regression of viral papillomas of left forelimb after therapy with topical imiquimod **(g)**.

On progress examination, 3 weeks later (13 weeks from initial presentation), the left forelimb swelling, nodules, and draining tracts improved markedly (Figure 2f), but the patient developed with acute diffuse swelling of the right carpus and several multifocal exophytic cutaneous masses were observed on the face and legs (Figure 2g). Radiographs revealed an aggressive, osteolytic bone lesion of the distal aspect of the right radius. Histopathological examination of the bone biopsy showed productive osteoblastic osteosarcoma. Fungal culture yielded no growth. Surgical removal of the cutaneous masses with histopathology revealed irregular epidermal hyperplasia with intranuclear keratinocyte inclusion bodies characteristic of viral-induced papillomas (Figures 3a–c). Immunosuppression with MMF (12 mg/kg) once daily was continued, prednisone and the previous therapies for *Nocardia* and *Curvularia* infections were discontinued.

About 4 weeks later, the patient presented with development of diffuse to coalescing viral-papillomas (VP) on the left forelimb (Figures 3d–f). Therapy with every other day topical imiquimod (5%; Perrigo; Allegan, MI, USA) resulted in near regression of the lesions 2 weeks later (Figure 3g). The patient was euthanized 3 weeks later (20 weeks from initial presentation) due to progression of osteosarcoma.

## Discussion

To the authors' best knowledge, this is the first case report of successful treatment of cutaneous opportunistic bacterial, fungal and viral infections in a canine patient with continued immunosuppressive therapy for IMHA. This case report confirms the increasing prevalence risk of opportunistic cutaneous infections in immunosuppressed dogs and the therapeutic challenges clinicians encounter when presented with these cases.

Ciclosporin administration is considered a significant risk factor for opportunistic invasive fungal infections (OIFI) in multi-immunosuppressor agent treatment protocols for immune-mediated diseases in dogs (3). Interestingly, prednisone, MMF, azathioprine, and leflunomide were not associated with the development of OIFI (3). The majority of dogs with OIFI are diagnosed with phaeohyphomycosis or hyalohyphomycosis; the most common genera of phaeohyphomycosis associated with OIFI includes *Curvularia* species (spp.), *Alternaria* spp., *Bipolaris* spp., *Cladosporium* spp., and *Paraconiothyrium* spp. (2, 3, 6). In this report, cutaneous OIFI affecting right forelimb was developed during 6-week multi-agent immunosuppressive protocol for IMHA and *Curvularia geniculata* was identified as a causative agent using next-generation sequencing from the fungal culture. Treatment of dogs that develop cutaneous OIFI, consists of discontinuation of immunosuppressive medications, administration of antifungal medications and surgical excision if lesions are solitary or on a distal extremity (3, 6). Discontinuing ciclosporin administration and reducing the dosage of prednisolone resulted in spontaneous cutaneous phaeohyphomycosis resolution in a single dog while IMHA was controlled in remission (9). However, in some patients, additional long-term systemic antifungal medications with discontinuation of immunosuppressive medications are needed for the successful OIFI treatment (3, 10). Importantly, systemic antifungal azoles (e.g., itraconazole, ketoconazole, fluconazole) should not be administered to OIFI patients unless ciclosporine is discontinued; systemic azoles increase blood levels of ciclosporine when jointly administered, which leads to more potent immunosuppression (11). Rapid clinical remission of cutaneous phaeohyphomycosis in our dog was achieved with discontinuation of ciclosporine and administration of systemic combination itraconazole and terbinafine; surgical approach and limb amputation were not needed. Combination antifungal therapy due to the severity of IMHA in our patient, systemic immunosuppression with MMF and prednisone was reduced but continued during the antifungal treatment.

Opportunistic bacterial and viral infections in dogs receiving MMF alone or together with glucocorticoids are not well-documented. The most common side effect in dogs during MMF and glucocorticoid administration for treatment of canine IMHA is diarrhea (12). Opportunistic cutaneous and systemic infections with *Nocardia* spp. in dogs have been associated with ciclosporine administration (1, 13). Nocardiosis can be difficult to diagnose due to the relatively slow growth of the organism and possible contaminants from tissue samples (1, 13). We removed the superficial part of the skin biopsy tissue to avoid surface skin contaminants in the culture in our dog. The use of MALDI-TOF MS, to identify most bacterial and fungal isolates, has revolutionized workflow and improved turnaround time in clinical microbiology laboratories. From 312 Nocardia isolates tested, 236 (76%) were correctly identified to the species level using MALDI-TOF MS (14). In our case, MALDI-TOF MS identified the cultured Gram-positive branching bacilli as *Streptomyces* spp; invasive *Streptomyces* infections are uncommon in animals and are sometimes considered to be contaminants (7). To confirm the MALDI-TOF MS bacterial identification results in our dog, 16S rRNA gene sequencing was performed on the bacterial isolate and revealed *Nocardia niigatensis*; 16S rRNA gene sequencing is commonly used for *Nocardia* identification as the 16S rRNA gene is highly conserved among *Nocardia* species (1). Identification of the *Nocardia* species as the causative pathogen, involved in our patient's skin lesions, is important because it allows different options for an effective systemic antimicrobial therapy. Taking into account the skin lesion severity and the time needed to obtain results from the bacterial culture, histopathology and gene sequencing, our patient was immediately started with oral doxycycline and enrofloxacin. Historically, TMS has been the antimicrobial drug of choice in dogs for its recognized activity against *Nocardia* spp. and low cost; TMS was not utilized in our IMHA patient due to concerns of a potential adverse effect of inducing hemolytic anemia (15). In this case, treatment with doxycycline and enrofloxacin resulted in significant clinical improvement of cutaneous nocardiosis lesions without adverse effects observed during treatment. Interestingly, tetracyclines are considered as an alternative oral agent for nocardiosis in humans with hypersensitivity to TMS or TMS refractory nocardiosis (16). Further studies are needed in both human and canine patients to provide additional antibiotic options for treatment of *Nocardia* infections.

Cutaneous PV infections can be developed in dogs without detectable immunodeficiency, are self-limiting and frequently regress spontaneously in one to 12 months (4, 5). Systemic immunosuppression has been associated with the development of PV-induced lesions in dogs (4, 5). Topical imiquimod is an immune modifier that acts by stimulating Th1 cytotoxic cell-mediated immune response (17). The reports of imiquimod usage in canine PV skin infections are limited; complete resolution of disseminated cutaneous viral papillomatosis with multimodal treatment involving surgical resection, topical imiquimod cream once daily and canine papillomavirus 2 vaccine was achieved in one dog (18). In our patient, surgical resection was not feasible with the location and severity of skin lesions. Topical imiquimod every other day resulted in significant regression of PV lesions within 2 weeks of therapy without side effects.

The patient developed osteosarcoma during the treatment of IMHA. In humans, IMHA is a known paraneoplastic syndrome but there is lacking veterinary literature linking cancer and IMHA in dogs, but cancer should be considered as a possible trigger to IMHA (19). Systemic immunosuppression with ciclosporine increases the risk of neoplasia in human transplant recipients with a case report of lymphoma in a canine patient treated with ciclosporine and ketoconazole for anal fistulas (19, 20). Our patient received ciclosporine for 6 weeks during initial treatment of IMHA, but was discontinued with development of an opportunistic fungal infection. In this case report, this breed is at an increased the risk of osteosarcoma development, but the role of immunosuppression in neoplasia development is not known (21, 22). The most recent literature has not reported immunosuppression as a risk factor for the development of osteosarcoma in canine patients (22).

## Conclusions

In conclusion, this case represents, to the best of our knowledge, the first description of the successful treatment of cutaneous opportunistic bacterial, fungal and viral infection in an immunosuppressed dog, even though the immunosuppressive drug treatments could not be discontinued. This case highlights the importance of monitoring multi-agent immunosuppressed patients for development of unusual opportunistic infections and pathogen identification with a deep skin biopsy, histopathology, tissue cultures and next-generation sequencing.

## Data Availability

The raw data supporting the conclusions of this manuscript will be made available by the authors, without undue reservation, to any qualified researcher.

## Ethics Statement

Best veterinary care was practiced in the clinical and diagnostic evaluation as well as treatments. The owner of the dog provided informed consent for all procedures prior to them being performed. As no experimental protocols were utilized, an institutional review was not required or performed.

## Author Contributions

ES and FB designed the concept of this case report. All authors contributed to the writing and figure generation and they approved the submitted version.

### Conflict of Interest Statement

The authors declare that the research was conducted in the absence of any commercial or financial relationships that could be construed as a potential conflict of interest.

## Abbreviations

IMHA: Immune-mediated hemolytic anemia
CBC: Complete blood count
MMF: Mycophenolate mofetil
H&E: Hematoxylin and eosin
GMS: Gomorimethenamine silver
PAS: Periodic cid-Schiff
PCR: Polymerase chain reaction
ITS: Internal transcribed spacer
ZN: Ziehl–Neelsen
MALDI-TOF MS: Matrix-absorption laser desorption ionization-time-of-flight massspectrometry
TMS: Trimethoprim-sulfamethoxazole
PV: Papillomavirus
OIFI: Opportunistic invasive fungal infections.

## References

[1] Yaemsiri S Sykes JE. Successful treatment of disseminated nocardiosis caused by Nocardia veterana in a dog. J Vet Intern Med. (2018) 32:418–22. 10.1111/jvim.14855 29105868 PMC5787162

[2] Dowling SR Webb J Foster JD Ginn J Foy DS Trepanier LA. Opportunistic fungal infections in dogs treated with ciclosporin and glucocorticoids: eight cases. J Small Anim Pract. (2016) 57:105–9. 10.1111/jsap.12367 25988822

[3] McAtee BB Cummings KJ Cook AK Lidbury JA Heseltine JC Willard MD. Opportunistic invasive cutaneous fungal infections associated with administration of cyclosporine to dogs with immune-mediated disease. J Vet Intern Med. (2017) 31:1724–9. 10.1111/jvim.14824 28887897 PMC5697195

[4] Lange CE Favrot C. Canine Papillomaviruses. Vet Clin Small Anim. (2011) 41:1183–95. 10.1016/j.cvsm.2011.08.003 22041210

[5] Munday JS Thomson NA Luff JA Papillomavirus in dogs and cats. Vet J. (2017) 225:23–31. 10.1016/j.tvjl.2017.04.018 28720294

[6] Dedeaux A Grooters A Wakamatsu-Utsuki N Taboada J. Opportunistic fungal infections in small animals. J Am Anim Hosp Assoc. (2018) 54:327–37. 10.5326/JAAHA-MS-6768 30272479

[7] Nicholls PK Allen G Irwin PJ. Streptomyces cyaneus dermatitis in a dog. Aust Vet J. (2014) 92:38–40. 10.1111/avj.12135 24471881

[8] Laurent FJ Provost F Boiron P. Rapid identification of clinically relevant Nocardia species to genus level by 16S rRNA gene PCR. J Clin Microbiol. (1999) 37:99–102. 9854071 10.1128/jcm.37.1.99-102.1999 PMC84177

[9] Dedola C Stuart APG Ridyard AE Else RW van den Broek AHM Choi JS Cutaneous Alternaria infectoria infection in a dog in association of therapeutic immunosuppression for management of immune-medicated haemolytic anaemia. Vet Dermatol. (2010) 21:626–34. 10.1111/j.1365-3164.2009.00875.x 20500496

[10] Swift IM Griffin A Shipstone MA. Successful treatment of disseminated cutaneous phaeohyphomycosis in a dog. Aust Vet J. (2006) 84:431–5. 10.1111/j.1751-0813.2006.00068.x 17156326

[11] Gray LL Hillier A Cole LK Rajala-Schultz PJ. The effect of ketoconazole on whole blood and skin ciclosporin concentrations in dogs. Vet Dermatol. (2013) 24:118–e28. 10.1111/j.1365-3164.2012.01064.x 23331687

[12] Wang A Smith JR Creevy KE. Treatment of canine idiopathic immune-mediated haemolytic anaemia. J Small Anim Pract. (2013) 54:399–404. 10.1111/jsap.12107 23879827

[13] Siak MK Burrows AK. Cutaneous nocardiosis in two dogs receiving ciclosporin therapy for the management of canine atopic dermatitis. Vet Dermatol. (2013) 24:453–e103. 10.1111/vde.12046 23781943

[14] Body BA Beard MA Slechta ES Hanson KE Barker AP Babady NE. Evaluation of the Vitek MS v3.0 Matrix-assisted laser desorption ionization-time of flight mass spectrometry system for identification of *Mycobacterium* and *Nocardia Species*. J Clin Microbiol. (2018) 56:1–12. 10.1128/JCM.00237-18 29643203 PMC5971548

[15] Trepanier LA. Idiosyncratic toxicity associated with potentiated sulfonamides in the dog. J Vet Pharmachol Therap. (2004) 27:129–38. 10.1111/j.1365-2885.2004.00576.x 15189298

[16] Wilson JW. Nocardiosis: updates and clinical overview. Mayo Clin Proc. (2012) 87:403–7. 10.1016/j.mayocp.2011.11.016 22469352 PMC3498414

[17] Stanley MA. Imiquimod and imidazoquinolones: mechanism of action and therapeutic potential. Clin Exp Dermatol. (2002) 27:571–7. 10.1046/j.1365-2230.2002.01151.x 12464152

[18] Levy BJ Sample SJ Yuan H. Multimodal treatment of a dog with disseminated cutaneous viral papillomatosis. Vet Dermatol. (2018) 29:78–e31. 10.1111/vde.12490 28921687

[19] Krisl JC Doan VP. Chemotherapy and Transplantation: the role of Immunosuppression in malignancy and a review of antineoplastic agents in solid organ transplant recipients. Am J Transplant. (2017) 17:1974–91. 10.1111/ajt.14238 28394486

[20] Blackwood L German AJ Stell AJ O'Neil T. Multicentric lymphoma in a dog after cyclosporine therapy. J Small Anim Pract. (2004) 45:259–62. 10.1111/j.1748-5827.2004.tb00233.x 15163054

[21] Simpson S Dunning MD de Brot S Grau-Roma L Mongan NP Rutland CS. Comparative review of human and canine osteosarcoma: morphology, epidemiology, prognosis, treatment and genetics. Acta Vet Scand. (2017) 59:71. 10.1186/s13028-017-0341-9 29065898 PMC5655853

[22] Makielski KM Mills LJ Sarver AL Henson MS Spector LG Naik S. Risk factors for development of canine and human osteosarcoma: a comparative review. Vet Sci. (2019) 6:48. 10.3390/vetsci6020048 31130627 PMC6631450

